# Relatedness modulates reproductive competition among queens in ant societies with multiple queens

**DOI:** 10.1093/beheco/arad004

**Published:** 2023-02-27

**Authors:** Heikki Helanterä, Martina Ozan, Liselotte Sundström

**Affiliations:** Faculty of Biological and Environmental Sciences, Organismal and Evolutionary Biology Research Programme, P.O.BOX 65, FI 00014, Helsinki University, Finland; Tvärminne Zoological station, J.A. Palménintie 260, FI 10900 Hanko, Finland; Faculty of Science, Ecology and Genetics Research Unit, FI 90014, University of Oulu, Finland; Faculty of Biological and Environmental Sciences, Organismal and Evolutionary Biology Research Programme, P.O.BOX 65, FI 00014, Helsinki University, Finland; Tvärminne Zoological station, J.A. Palménintie 260, FI 10900 Hanko, Finland; Faculty of Biological and Environmental Sciences, Organismal and Evolutionary Biology Research Programme, P.O.BOX 65, FI 00014, Helsinki University, Finland; Tvärminne Zoological station, J.A. Palménintie 260, FI 10900 Hanko, Finland

**Keywords:** conflict, cooperation, *Formica fusca*, inclusive fitness, kin discrimination, reproductive skew

## Abstract

Reproductive sharing in animal groups with multiple breeders, insects and vertebrates alike, contains elements of both conflict and cooperation, and depends on both relatedness between co-breeders, as well as their internal and external conditions. We studied how queens of the ant *Formica fusca* adjust their reproductive efforts in response to experimental manipulations of the kin competition regime in their nest. Queens respond to the presence of competitors by increasing their egg laying efforts, but only if the competitors are highly fecund and distantly related. Such a mechanism is likely to decrease harmful competition among close relatives. We demonstrate that queens of *Formica fusca* fine-tune their cooperative breeding behaviors in response to kinship and fecundity of others in a remarkably precise and flexible manner.

## Teaser

Ant societies may contain multiple queens competing over reproduction. In the ant, *Formica fusca*, queens adjust their egg laying effort to kinship of their nestmates. Our experiments show that queens increase their egg laying rate when exposed to odors of a highly fecund second queen, but only when this queen is not close kin. When exposed to odors of a highly fecund close relative, queens decrease their egg laying in order to decrease competition.

## INTRODUCTION

Reproductive cooperation often involves individuals with diverging fitness interests, so that reproductive conflicts among them arise. A multitude of ecological, genetic, and social factors have been proposed to shape the outcome of reproductive conflicts and the relative reproductive success of cooperatively breeding individuals (i.e., reproductive skew; Reeve and Keller 2001). The strategies used for increasing reproductive shares depend on the kinship between group members, differences in their reproductive traits, and the availability of information about these. For example, in dwarf mongooses, *Helogale parvula*, the dominant female inhibits ovulation of subordinates via aggression (Creel et al. 1992), by which she is able to almost entirely monopolize reproduction in the group. Conversely, competitive reciprocal destruction of each other’s eggs by females of the cooperatively breeding acorn woodpecker, *Melanerpes formicivorus*, equalizes reproductive shares (Koenig et al. 1995), believed to promote synchrony in egg laying and reduce reproductive skew. Such interactions may be modulated by relatedness in complex ways, such as when dominant banded mongoose, *Mungos mungo*, females preferentially harass and even evict close rather than distant kin, as close kin are less likely to aggressively retaliate (Thompson et al. 2017).

In eusocial insects, reproductive division of labor between morphologically distinct castes permits direct fitness to only a minority of individuals, that is, the queen(s), whereas the majority of individuals, that is, the non-reproductive workers, largely only gain indirect fitness returns (Hamilton 1964). Colonies of many ant species furthermore contain several reproductively active queens (polygyny), which typically leads to reduced per capita fecundity compared to those in single-queen colonies of the same species (van der Meer et al. 1992; Trunzer et al. 1998; Schrempf et al. 2011; Yamamoto and Matsuura 2011). Reproductive conflicts may arise among co-breeding queens owing to limited colony resources (Ross 1988; Fournier et al. 2004; Bargum and Sundström 2007a; Kümmerli and Keller 2007). The reproductive shares of queens may be unequal (Hannonen and Sundström 2002; Fournier et al. 2004), but this has usually been assessed only at the pupal stage, which reflects the final outcome of reproductive competition (but see Ross 1988). Thus, the question at what stage such differences arise, and which factors affect the shares at the early stages, remains uncharted.

The reproductive shares of queens may be affected by several factors. First, queens may differ in intrinsic traits, such as age, fecundity, and egg viability (Hughes and Strassmann 1988; Ross 1988; Hannonen and Sundström 2002; Holzer et al. 2006). The shares may also reflect temporal variation in fecundity, whereby queens that start oviposition early gain larger shares of reproduction to the detriment of late starters (Ozan et al. 2013). Second, queens may compete over reproduction via overt aggression or brood cannibalization. The former may be rare in secondarily polygynous ant colonies, that is, those where multiple queens co-exist in mature colonies (Keller 1993), but the latter has been observed in, for example, *Ectatomma tuberculatum* (Hora et al. 2007), and *Leptothorax acervorum* (Bourke 1994). Third, queens may chemically suppress oviposition of other queens, so that the presence of a competing queen or her odors decreases fecundity, as found in both ants (Vargo 1992; Holman et al. 2013; Abril and Gómez 2020), and termites (Yamamoto and Matsuura 2011). Finally, indirect genetic benefits mediated by relatedness patterns may influence interactions among nest mate queens (Hamilton 1964). In secondarily polygynous ant colonies, the relatedness among queens can vary extensively (Stuart et al. 1993; Brown and Keller 2002; Hannonen and Sundström 2003; Kikuchi et al. 2007; Vásquez and Silverman 2007; Meunier et al. 2011). This may create an incentive for queens to adjust their responses to competing queens and so balance direct fitness gains from own reproduction with indirect fitness gains via related queens. Indeed, inclusive fitness logic predicts that the ability to facultatively respond to kinship is more likely to be found in situations where kinship among group members varies, as has been shown to be the case in cooperatively breeding vertebrates (Cornwallis et al. 2009).

In this study, we carry out experiments to tease apart the different factors that contribute to reproductive sharing among nestmate queens in the ant *Formica fusca.* Colonies of this species often have several queens, with variable degrees of relatedness (Hannonen and Sundström 2003). The queens do not compete by overt aggression, yet differ considerably in both fecundity, and their ultimate reproductive shares (Hannonen and Sundström 2002; Ozan et al. 2013). We investigate whether queen fecundity changes in response to the presence of odors of another queen, and whether the response depends on the fecundity of, and relatedness to the other queen. We predict that under high relatedness, queens trade indirect fitness benefits in favor of potentially costly reproductive competition and respond to highly fecund nestmates with decreased egg laying. Conversely, under low relatedness the presence of a fecund competitor would trigger increased egg laying as an effort to increase direct fitness.

## METHODS

### Colony collection and maintenance

We collected colonies of the black ant, *Formica fusca*, on the Hanko peninsula, southwestern Finland in late April and early May 2012. The colonies reside in rotting tree stumps and logs, under rocks and in soil, and in the spring, when queens are warming near the surface, entire colonies, or at least a large majority of the workers and queens, can be collected. The colonies were sorted to determine queen number, and to ensure that oviposition had not yet commenced, after which they were stored at +4 °C with nest material and peat for 1–2 weeks, until the experiments started. These conditions emulate continued hibernation and prevent queens from resuming reproduction before the onset of experiments. Once enough colonies were available for the experiments, the ants were transferred to ambient temperature (22–24 °C) to prepare them for the experiments. At this stage, the nests were housed in plastic trays (30 × 40 × 15 cm) with plaster bottom, and the walls coated with Fluon^TM^. Water was provided in Eppendorf tubes plugged with cotton, and the ants were fed standard Bhatkar diet (Bhatkar and Whitcomb 1970) daily.

### Experimental design

**Figure 1.**
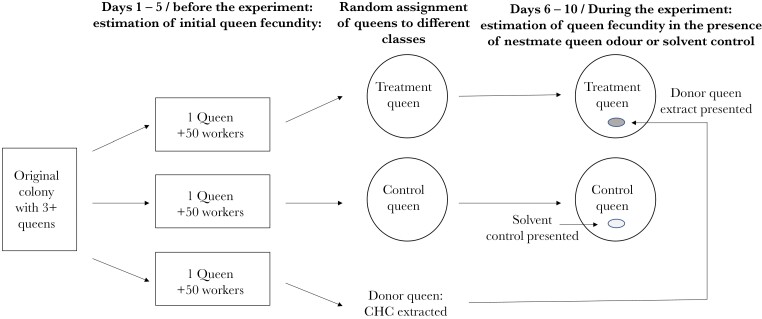
The experimental set-up for assessing queen fecundity in the presence of a nest mate queen odor. Following oviposition in the presence of workers (square boxes), the cuticular hydrocarbons of the donor queens were extracted. The two remaining queens were placed in isolation overnight, and then assigned to their experimental treatments (circles). The treatment queen was housed with a glass bead coated with the CHC of the donor queen and the control queen was housed with a glass bead treated with pentane control.

To investigate whether queen fecundity changes in response to chemical cues that signal the presence of another queen, we used colonies with three or more queens (average = 7.6, SD = 6.7) in the experiment. The experiment comprised triplets of nestmate queens, the fecundity of which was assessed at the start, after which the three queens were randomly assigned to one of three roles: Two queens continued in the experiment as treatment and control queens, respectively, and the third served as a source of odors (donor queen), to be presented to the treatment queen (Figure 1).

We set each of the three queens up in a separate experimental nest with an entourage of ca 50 workers (Figure 1). After 5 days, we counted the number of eggs each queen had laid, to assess their fecundity. We then killed the donor queen by freezing and extracted the non-volatile cuticular compounds, to be used as chemical cues for the presence of the treatment queen in the colony. The whole-body surface chemicals of the donor queen were extracted in 200 µL Chromasolv pentane (Sigma-Aldrich). The first extraction volume was allowed to evaporate and the dissolved chemicals re-diluted in 160 µL pentane. After extracting the cuticular compounds, the donor queens were placed in 94% ethanol for genotyping.

The two remaining queens were used in the experiment to assess how the odor of a donor queen with either high or low fecundity influences the fecundity of the treatment queen. The queens were first placed individually on Petri dishes without workers overnight before the experiment started (Figure 1). This was done because we wanted to make sure that any effects or worker attention would not carry over to the experiment. The experiment started by placing the queens on their own in plastic jars (Ø 7 cm), lined with plaster, and the walls coated with Fluon™. A cavity (Ø 3 cm) covered with a piece of transparent red plastic sheet (2 × 5 cm) served as a brood chamber. An oval-shaped glass bead (8 × 5 mm) treated with either 10 µL of extract from the donor queen (treatment queens) or pentane (control queens), was placed in the brood cavity to simulate the presence of another queen (Figure 1). The glass beads were treated with 10 µL of extract (treatment colonies) or solvent (control colonies) every 12 h for the duration of the experiment (5 days). The queens received water, as described above, but no food during the experiment. At the end of the experiment, we counted the eggs the queens had laid in the container, to determine queen fecundity during the experiment, in the absence of workers, and placed the treatment queens in 94% ethanol for genotyping. The counts were done blindly, as the person counting the eggs was not aware of the origin of the extract given to each queen (solvent control or competitor queen treatment), as the vials were relabeled for the duration of the experiment, nor the relatedness between queens (not yet analyzed at this stage).

To assess whether donor queen relatedness influences queen fecundity we genotyped the donor and treatment queens, as well as eight workers per nest. We did not genotype the control queens, as these were not exposed to the odors produced by another queen and therefore had no target for comparison. We used 12 polymorphic microsatellite loci designed for *Formica* species: FL12, FL20, FL21 (Chapuisat 1996); FE13, FE16, FE19, FE21, FE42, and FE51 (Gyllenstrand et al. 2002) and FY4, FY7, FY13 (Hasegawa and Imai 2004). Details of the protocols are given in the Supplementary Material.

### Statistical analyses

We estimated the pairwise relatedness among treatment and donor queens and the eight workers with the maximum likelihood estimation in the software ML-Relate (Kalinowski et al. 2006). To investigate whether the presence of a nest mate queen (represented by the odor of the donor queen) influences queen fecundity, we first tested the difference in the number of eggs laid between the treatment queen and the control queen in each colony in the experimental phase using paired *t*-test. We then tested whether the fecundity of the donor queen affects the fecundity of the treatment queen, and whether relatedness between donor and treatment queens, or the average relatedness structure of the colony modify this effect, using a linear model in R (R Development Core Team 2014). Our continuous predictors were the fecundity of the donor queen and three different measures of relatedness, each included in a separate model: relatedness between the treatment and donor queen (hereafter *r(q)*), the average of all pairwise relatedness estimates among workers in the colony (*r(w)*), and average of pairwise relatedness among all genotyped individuals (the two queens and eight workers, *r(all)*). As the fecundities of treatment and control queens in the same nest were correlated (see results), we also needed to account for this dependence in our analyses. Thus, we used as our response variable the residual of the correlation between the fecundities of treatment and control queens within colonies. This factors out overall differences among colonies and measures the fecundity differences between queens within colonies. Any consistent differences in this measure reflect the effects of treatment, so that a positive value indicates a relatively high fecundity of the treatment queen, presumably caused by the treatment. We also carried out additional analyses to investigate the scenario that queen responses to the fecundity of a competitor would be driven by average relatedness in the colony rather than relatedness among queens. We found no support for this scenario (see Supplementary Material).

## RESULTS

The relatedness between treatment and donor queens was highly variable (*r(q)* average = 0.63, SD = 0.27, range from 0.04 to 1), whereas relatedness among workers was less variable, and lower (*r(w)* average = 0.33, SD = 0.08, range from 0.21 to 0.4). Values of *r(q)* and *r(w)* of each colony were not correlated (*r* = −0.1, *n* = 15, *P* = 0.72).

The average of eggs laid per day for all queens before the experiment (in the presence of workers) was 19.6 ± 11.0 (mean ± SD), and did not vary between sets of queens assigned to different classes (queens assigned as donors 19.7 + 11.1, treatment 20.0 + 11.7, and control 19.1 + 10.8). During the experiment, when workers were absent, daily fecundity for the two types of queens used in the experiment (treatment and control) decreased to 2.3 ± 1.9 (mean ± SD). The daily egg-laying rates of the treatment and the control queens during the experiment did not differ significantly from each other (treatment queens 2.5 ± 2.1, control queens 2.0 ± 1.7, paired *t*-test: *t* = 1.51, df = 14, *P* = 0.153), but were correlated within colonies (Spearman’s *r* = 0.59, *P* < 0.001). This suggests that queen fecundity was unaffected by the presence versus absence of cuticular hycrocarbon (CHC) odor of a nest mate queen.

**Figure 2.**
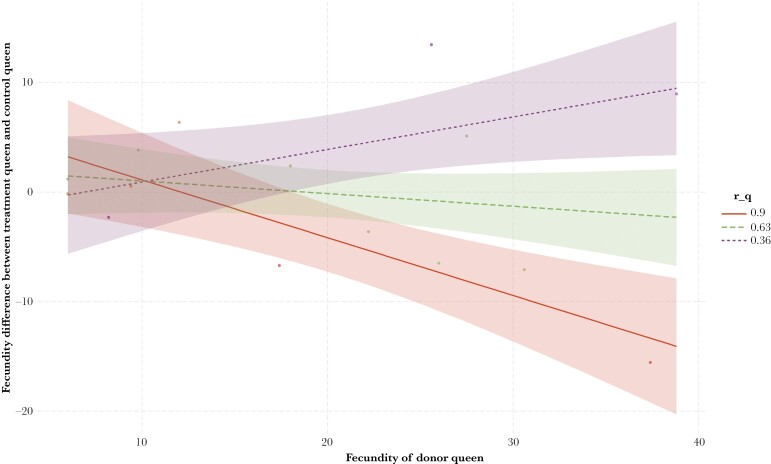
The effect of fecundity of the donor queen, on the fecundity difference between treatment and control queens as a function the relatedness between treatment and donor queens. The slopes (and 95% CI) of the relationship between predicted queen fecundity and the fecundity of her nest mate were estimated at high (mean + 1SD), mean and low (mean − 1SD) relatedness, using a simple slopes analysis (Dawson 2014), using R-package *jtools* (Long 2020).

The fecundity of the treatment queen was lower compared to that of the control queen when the fecundity of the donor queen was high, and if the pairwise relatedness between treatment and donor queens was high. This relationship was reversed when the relatedness between the treatment and the donor queen was low (fecundity: *t* = 3.5, *P* = 0.005; *r(q)*: *t* = 1.8, *P* = 0.09; fecundity × *r(q)*: *t* = −4.3, *P* = 0.001; df = 11, *r*^2^ = 0.73; Figure 2). No significant effects of donor fecundity, relatedness, or their interaction were observed when relatedness was measured as the average worker relatedness or the average relatedness among all genotyped individuals (model with *r(w)*: fecundity: *t* = −1.3, *P* = 0.22; *r(w)*: *t* = −0.9, *P* = 0.40; fecundity × *r(w)*: *t* = 1.2, *P* = 0.25; df = 11, *r*^2^ = 0.15, model with *r(all)*: fecundity: *t* = −0.66, *P* = 0.52; *r(all)*: *t* = −0.58, *P* = 0.58; fecundity × *r(all)*: *t* = 0.55, *P* = 0.59; df = 11, *r*^2^ = 0.05).

## DISCUSSION

We tested whether the fecundity of co-breeding queens varies in response to chemical cues from competitors. We show that the response to competitor odors was modulated by both fecundity and kinship. Highly fecund competitors had a negative effect on treatment queen fecundity when the queens were close relatives, but this effect was reversed when relatedness was low. This suggests that highly fecund queens are not able to simply coerce diminished egg laying in their competitors, but that queens respond to chemical signals from their nestmates in a facultative, kinship sensitive manner.

A kinship sensitive effect of the odors of another queen stands in contrast to observations from other social insects where the presence of a competing queen or her odors simply decreases fecundity (Vargo 1992; Holman et al. 2010; Yamamoto and Matsuura 2011; Abril and Gómez 2020 but see Bourke 1993, 1995). The more complex facultative and kinship dependent response observed here in *F. fusca* could reflect the highly variable kin structure of the species, where multiple-queen colonies commonly contain a mixture of close and distant relatives, even when the average relatedness is high (Hannonen et al. 2004; Bargum et al. 2007b; Ozan et al. 2013). In such conditions, facultative adjustments can lead to inclusive fitness gains (Cornwallis et al. 2009), in contrast to, for example, the multiple-queen colonies of *Solenopsis invicta* (Vargo 1992) or *Linepithema humile* (Abril and Gómez 2020)), or foundress associations of *Lasius niger* (Holman et al. 2013), in which relatedness is more uniformly very low or even zero. Our results show similarities to those found in allodapine bee *Exoneura robusta*, in which queens inhibited their ovary development more in when in associations with related queens than with unrelated queens (Harradine et al. 2012). Such findings suggest that the chemical signals between queens may be mutually beneficial rather than coercive in nature, as in the honest signals that mediate effects of a queen on worker fertility (Holman et al. 2010b). Thus, under high relatedness not all queens need to lay eggs at a very high rate in order to maximize colony productivity, and in the presence of a highly fecund close relative a queen might increase her inclusive fitness by decreasing current reproductive efforts.

Our results raise the question why queens should trade-off direct reproduction for indirect in the presence of related queens. Possible reasons include avoidance of indirect fitness costs of competition with kin (West et al. 2002), and direct costs of sub-optimally high reproductive investment on future reproduction or survival. As such direct costs have not been observed in ants in recent studies (Heinze and Schrempf 2012; Heinze et al. 2013; von Wyschetzki et al. 2015), we suggest that reproductive allocation may be optimized at the colony, rather than individual level, in a high relatedness setting.

It is possible, for example, that the queens who decrease their current egg laying in the presence of fecund close kin, are able to lay more eggs later in the season to increase production of future workers in the nests. This might benefit all co-habiting queens, and pay off under high kinship, as has been observed in this species (Bargum and Sundström 2007a; Bargum et al. 2007b). The experiment was carried out early in the season when the eggs that are reared into sexuals are being laid, and it is known that the reproductive shares of the queens differ between sexual and worker brood (Bargum and Sundström 2007a). However, it remains to be studied whether the processes here observed result in productivity benefits to colonies where closely related queens avoid excessive competition.

Our results suggest that *F. fusca* queens respond facultatively to kinship associated odor cues and so come to enhance their indirect fitness returns. While the odors on queen cuticle have not been analyzed in *F. fusca*, eggs laid by queens have been shown to carry matriline-specific chemical cues in this and other *Formica* species (Helanterä and d’Ettorre 2015). Furthermore, workers are able to recognize their mother even when reared in foster nests (El-Showk et al. 2010), and to nepotistically favor closely related queens (Hannonen and Sundström 2003). Together, these findings suggest that queens could be able to use chemical information to detect both the fecundity and kinship of odors presented to them, even if such kin discrimination within nests is rare in insect societies (Boomsma and d’Ettorre 2013).

Alternatively, how a queen responds to a competitor’s fecundity could be mediated by the average relatedness of the colony, in addition or instead of the pairwise relatedness of the individuals. This would not necessitate the ability to assess relatedness directly but could rely on assessing the genetic diversity of the nest more generally. Such facultative responses to colony kin structure have been observed in the context of sex ratio manipulation by workers in *Formica* ants (Sundström 1994; Sundström et al. 1996; Boomsma et al. 2003), but not in interactions among the queens. Our current data did not show an effect of average relatedness or genetic diversity among colony members on queen fecundity in *F. fusca*, but this remains an important scenario to explore.

We show that despite their dependence on non-reproductive helpers for survival and brood care, queens can nevertheless mediate their reproductive interests by subtle means when reproduction is shared. Thus, while workers of the species have their means of affecting reproductive shares in a nest via nepotism (Hannonen and Sundström 2003) and their own direct reproduction (Ozan et al. 2013), also queens make facultative decisions to affect reproductive shares. These responses are non-aggressive and likely mutually benefit both queens and workers when they are close kin. *Formica fusca* queens are able to achieve this by responding facultatively to kin informative chemical cues on nest mate cuticle to increase inclusive fitness returns once queen begins to oviposit. Facultative reproductive adjustments based on relatedness have likewise been reported in cooperatively breeding vertebrates, especially in conditions where groups comprise a mixture of close and distant relatives (Cornwallis et al. 2009), where inclusive fitness benefits of discrimination are likely to be high. Such responses are less commonly observed in social insects (but see Foster and Ratnieks 2000; Harradine et al. 2012; Ozan et al. 2013), and even more rarely among co-breeding queens. The ability of *Formica fusca* queens to actively shape their own reproductive effort before the onset of brood rearing suggests that queens in associations with apparent lack of overt reproductive conflicts have more power over their own reproductive destiny than may be generally appreciated, and that the balance between cooperation and conflict is fine-tuned to the kin structure context and the individual traits of the co-breeders.

## Supplementary Material

arad004_suppl_Supplementary_Material

## Notes

We thank Kim Jaatinen for helpful comments on an earlier manuscript version.
