# Supplementary material for: Relatedness modulates reproductive competition among queens in ant societies with multiple queens
Source: Behav Ecol. 2023 Feb 27;34(3):340–5. doi: 10.1093/beheco/arad004 (PMC10183207; doi:10.1093/beheco/arad004)
Supplement: arad004_suppl_Supplementary_Material [file arad004_suppl_supplementary_material.pdf]

Supplementary information

Supplementary methods: DNA extraction and PCR methods

DNA was extracted by incubating two legs from each queen or worker O/N at 56°C in 2.5:100µL proteinase K-Chelex (Schultner et al. 2014). PCRs were run in 10 µL reactions using 5 µL Qiagen Type-It microsatellite multiplex buffer, 3 µL deionized water, 1 µL optimized primer mix, and 1 µL DNA at 1:200 dilution on a 3730 ABI sequencer, following the protocols recommended by Qiagen. The microsatellite peaks were scored with Genemapper v. 4.1, and allele calling confirmed manually.

Supplementary analyses: effects of average relatedness on queen fecundity

In order to investigate the scenario that queen fecundity variation would be driven by average relatedness of the nest, and apply to queens independent of their treatment/control status, we analysed three linear models, using the lm command in R. Each of them had as the response variable the daily average number of eggs laid by the queen during the experiment (i.e. in the absence of workers, while being exposed to donor queen odours (treatment queens) or solvent control (control queens)). As explanatory variables we used treatment/control status, a relatedness measure and their interaction. The three models differed only in the relatedness measure used.

The first model had r(all) as the relatedness measure (i.e. average of pairwise relatedness estimates among the eight genotyped workers and the treatment and donor queens). None of the explanatory variables had significant effects on queen fecundity (Treatment: $t = 0.4$, $p = 0.69$, r(all): $t = 1.4$, $p = 0.17$, interaction: $t = -0.19$, $p = 0.85$).

The second model had r(q) as the relatedness measure (i.e. pairwise relatedness between the treatment and donor queens). None of the explanatory variables had significant effects on queen fecundity (Treatment: $t = 1.5$, $p = 0.14$, r(q): $t = 0.11$, $p = 0.91$, interaction: $t = -1.3$, $p = 0.20$).

The third model had r(w) as the relatedness measure (i.e. average of pairwise relatednesses among the eight genotyped workers). Relatedness r(w) had a positive effect on egg laying, but treatment and the interaction term did not (Treatment: $t = 0.05$, $p = 0.96$, r(w): $t = 2.2$, $p = 0.04$, interaction: $t = 0.18$, $p = 0.86$).

Thus, we find no support for the scenario that treatment queens would respond to nestmate odours based on the relatedness structure of their natal nest on average rather than their pairwise relatedness to the donor queen. In such a case we would have expected to observe an interaction where relatedness has a positive effect on the fecundity of treatment queens (exposed to nestmate odours) but not for control queens (exposed to solvent only, and thus likely to behave as in the absence of nestmate queens).

Supplementary Information: description of data files found at doi:10.5061/dryad.r2280gbh9

total_egg_laying_R1.txt

Variables:

colony: ID of the original field collected colony where queens and workers originated from
queen: running ID of the queen
role: whether the queen was a treatment or a control queen

workers: absent: fecundity in the absence of workers, during the experiment, present: fecundity in the presence of workers, before the experiment
egg-laying: daily number of eggs laid
r_all: average of pairwise relatedness estimates among the eight genotyped workers and the treatment and donor queens
r_q: pairwise relatedness between treatment and donor queen
r_w: average of pairwise relatedness estimates among the eight genotyped workers
q1pre: total number of eggs laid by the treatment queen of the colony in the presence of workers, before the experiment
q2pre: total number of eggs laid by the control queen of the colony in the presence of workers, before the experiment
q3pre: total number of eggs laid by the donor queen of the colony in the presence of workers, before the experiment q1post: number of eggs laid by treatment queen under exposure to odours from donor queen
q2post: number of eggs laid by treatment queen under exposure to solvent
